# Average direct cost of peripherally inserted central catheterization by nurses in hospitalized patients: a cost estimate

**DOI:** 10.31744/einstein_journal/2024GS0982

**Published:** 2024-11-19

**Authors:** Maithê Gomes Lima Zandonadi, Danielly Negrão Guassú Nogueira, Amanda Salles Margatho do Nascimento, Paula Buck de Oliveira Ruiz, Natália Marciano de Araújo Ferreira, Suellen Karina de Oliveira Giroti, Flávia Meneguetti Pieri

**Affiliations:** 1 Universidade Estadual de Londrina Londrina PR Brazil Programa de Pós-Graduação em Enfermagem, Universidade Estadual de Londrina, Londrina, PR, Brazil.; 2 Universidade de São Paulo de Ribeirão Preto Escola de Enfermagem Ribeirão Preto SP Brazil Escola de Enfermagem, Universidade de São Paulo de Ribeirão Preto, Ribeirão Preto, SP, Brazil.

**Keywords:** Nurses, Catheterization, Peripheral, Vascular access devices, Cost analysis, Direct service costs

## Abstract

**Objective:**

This study aimed to estimate the direct costs of peripherally inserted central catheterization by nurses for hospitalized patients.

**Methods:**

A cost estimation study using a quantitative approach was conducted in a public teaching hospital in northern Paraná to calculate the direct costs of peripherally inserted central catheterization. The population included all medical records of patients between 15 and 99 years of age who were hospitalized and underwent peripherally inserted central catheterization by nurses between January 1, 2019, and December 31, 2021, totaling 664 insertions. The sample comprised 631 insertions.

**Results:**

The catheter kits (epicutaneous catheter + introducer + angulators) corresponded to the items with the highest unit costs and the greatest impact on the composition of costs. The cost of peripherally inserted central catheterization was US$ 217.14 (SD=75.21), with the cost of materials and staff's labor being US$ 195.39 (SD=74.15) and US$ 20.00 (SD=2.22), respectively.

**Conclusion:**

Materials represented the highest cost, which was explained by the high unit cost of catheters and kits used in echocardiography, followed by the staff's labor costs. The estimated average direct cost allowed for financial visibility of the inputs used. The key challenge is promoting lasting changes in the behavior of managers who carry out administrative functions in healthcare institutions, where proper budget management directly affects the allocative efficiency of resources and the quality of care.

## In Brief

This study demonstrates that materials represent the highest cost with respect to the placement of peripherally inserted central catheters by nurses in hospitalized patients, which is explained by the high unit cost of the catheter kits used in echocardiography, corresponding to the items with the highest unit cost and the greatest impact on the composition of costs, followed by the staff’s labor costs.

## Highlights

▪ The highest cost is for materials, followed by the staff’s labor costs.
▪ The total cost of peripherally inserted central catheterization is US$ 217.14.
▪ Measuring the estimated average direct cost allows financial visibility of the inputs used.

## Graphical abstract

**Figure:**
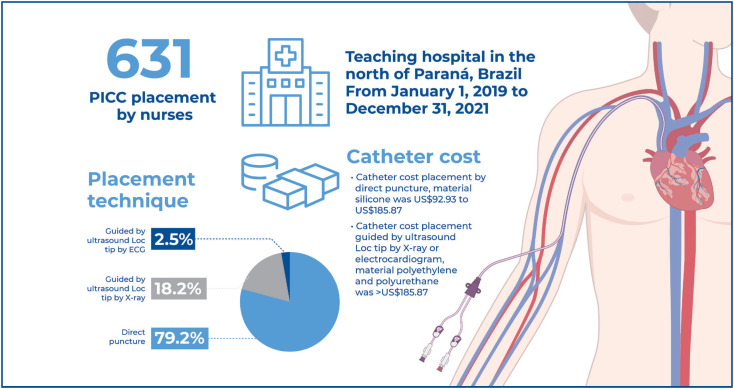

## INTRODUCTION

The peripherally inserted central catheter (PICC), a device inserted through a centrally located peripheral vein, is considered a safe form of venous access in various care settings to support intravenous therapy (IVT).^(1,2)^

As the placement of PICC by nurses requires attention to good practices, continuing education, and technical expertise, its operationalization is dependent on the financial and educational support of the professionals involved as well as the availability of resources by healthcare institutions.^(3-6)^

In Brazil, there has been a gradual increase in the use of PICC. In other countries, such as the United States, its use is widespread, with significant figures of approximately three million PICC placements per year.^(5,7,8)^

Regarding technical and legal attributions, in Brazil, competence in placing PICCs is conferred on the nurse after professional qualification and/or training, in line with the Resolution of the Federal Nursing Council (COFEN - *Conselho Federal de Enfermagem*) No. 258/2001.^(9)^

Since the device insertion requires qualified human resources and specific material resources and technologies, which impose additional costs on institutions, the inherent costs should be properly determined and managed.^(3)^

In this context, nurses play a leading role in the handling and management of the resources involved in the procedure and stand out in managerial actions within healthcare institutions. Their tasks include enhancing feasibility and effective utilization of available resources in care activities.^(10,11)^

The analysis of health costs has been a subject of heated debate because of the importance of calculating these costs for healthcare institutions. However, few studies have investigated the costs associated with PICC placement, including direct costs, which shows a gap in knowledge on the subject.^(12)^

To understand the costs involved in PICC placement, this study was conducted to support the decision-making process and adherence to practices that guarantee quality patient care, promote the allocative efficiency of the resources involved, and enhance the financial sustainability of healthcare institutions.

## OBJECTIVE

This study aimed to estimate the direct costs of peripherally inserted central catheter placement by nurses in hospitalized patients.

## METHODS

This quantitative cost estimation study attempted to determine the average direct cost (ADC) of PICC placement.

This study was conducted at a public teaching hospital in Northern Paraná, Brazil. This university hospital is academically linked to the Health Sciences Center (CCS) of the *Universidade Estadual de Londrina* (HU-UEL). This institution has 451 beds distributed among the inpatient units, emergency rooms (ER), and intensive care units (ICU) for adults, children, and neonates. It serves approximately 250 municipalities in Paraná and more than 100 cities in other states. The hospital was selected as the setting for this study because of its significance as a strategic and traditional reference center for the Unified Health System (SUS - *Sistema Único de Saúde*) in the region, with a high volume of care, and because it has good nursing practices combined with the use of vascular devices by the IVT team.

The ADC analyzed in this study refers to the costs directly involved in the PICC placement process and, by definition, are the calculations of the products that are measurable without the need for apportionment.^(13,14)^ These costs were measured from the perspective of hospital managers.

The methodological framework followed the recommendations of the Consolidated Health Economic Evaluation Reporting Standards (CHEERS) to standardize and promote transparency and methodological rigor.^(15)^ In compliance with ethical precepts, this project was approved by the Human Research Ethics Committee of the *Universidade Estadual de Londrina* (CAAE: 58785122.0.0000.5231; #5.439.281), and the researchers signed a confidentiality and nondisclosure agreement.

To establish the population, we considered all the medical records of hospitalized adolescent and adult patients aged 15-99 years who underwent PICC placement by nurses from January 1, 2019, to December 31, 2021, totaling 664 insertions. The study sample was selected using convenience sampling.

All PICC procedures performed by nurses were included. Meanwhile, all procedures performed by medical professionals in adult inpatient and intensive care units were excluded. Medical records with incomplete and/or blank data, without the possibility of retrieving information, were considered losses.

Of the 664 procedures performed, a sample of 631 insertions was included in the study. Thirty-three procedures were considered losses due to incomplete data in the medical records.^(16)^ Insertions that took place in neonatal and pediatric units, considering neonates up to children under 14 years, 11 months, and 29 days, were not part of the scope of this research.

At the time of the analysis, the institution had 28 nurses qualified to place PICC, 7 of whom were residents, and 21 were permanent staff, as well as a nursing technician who acted as a technical assistant during the procedure. As they all had similar expertise, the length of their professional experience was not regarded as an inclusion variable.

Residents participate in UEL's residency programs, which are linked to teaching hospitals in various departments, including adult intensive care, urgency and emergency, infectology, and obstetric nursing. It should be noted that the institution considers a professional to be qualified to place the device if, after undergoing specific training, they have placed approximately five catheters under the supervision of another qualified nurse.

The study was conducted in three stages (Table 1). The first step was the identification of activities related to PICC placement through process mapping.

**Table 1:** Protocol for conducting the study on peripherally inserted central catheter placement by nurses

**Step 1: Process mapping**		
**Objective**		**Developments**
Understand the flow of activities related to PICC placement in force in the institution		Interviews with the intravenoustherapy team (IVT) Validate the PICC placement process through the current standard operating procedure (SOP) with the information passed on by the IVT team
Identify the departments involved in obtaining the direct labor cost (DLC) of professionals		Contact the departments involved to obtain the variables for the composition of the DLC
Identify the departments involved in the value chain of the materials/medicines/solutions/imaging tests used in PICC placement		Contact the departments involved and survey the costs of materials/medicines/solutions/imaging tests.
Map the PICC placement		Create a flowchart
**Step 2: Data collection**		
**Objective**	**Developments**	
Develop instruments for data collection and storage	Record the variables collected in an electronic spreadsheet	
Collect data regarding the number of materials used and time (duration) of the procedure	Data collection through an electronic questionnaire that was made available to the IVT team	
Collect demographic, clinical, therapeutic, and catheter data	Data collection through the electronic spreadsheets provided by the IVT team	
Validate the demographic, clinical, therapeutic, and catheter data, collected from the electronic spreadsheets	Verification of electronic medical records (Medview) for validation of demographic, clinical, and therapeutic data related to PICC	
Collect data on the salaries of professionals who enter the PICC to obtain the unit cost of the professionals’ DLC	Collection of professionals’ salaries in the transparency and validation portal with the HR department	
Raise material/drug/solution and catheter costs	Collection of the costs of materials/medicines/solutions through SICOR in the materials advisory department	
Raise the costs of the imaging test for confirmation of the tip of the PICC	Collection of imaging costs through the SIGTAP table and perform validation with the radiology department	
**Step 3: Obtaining the adc, data analysis, and conclusion**		
**Objective**	**Developments**	
Calculate the professionals’ DLC who insert the PICC	Multiply the time spent (duration) by the nurse and the technical assistant by the unit cost of the DLC and add it to the total ADC of PICC placement	
Calculate the unit cost and total cost of all inputs used to place the PICC	Multiply the unit cost of inputs by the average amount of each input used and then add the total cost of inputs for later inclusion in the total sum of ADC	
Calculate the cost of X-ray	Add X-ray cost to the totalADC of PICC placement	
Verify the correlations between the analyzed variables and the degree of significance	Submit the data to the relevant statistical tests through the SPSS Statistics Software (version 23) and linked Excel tool features (version 2016)	
Analyze categorical variables by absolute and relative numbers	Submit the data to the relevant statistical tests through the SPSS Statistics Software (version 23) and linked Excel tool features (version 2016)	
Present the demographic, clinical, and therapeutic profiles as well as the characteristics of inserted catheters and the ADC of PICC placement by nurses	Synthesize the results obtained and disseminate them later in the institution	

PICC: peripherally inserted central catheter; SICOR: Integrated Purchasing and Budgeting system; SIGTAP: Procedure Table Management System.

The second stage involved data collection. Demographic, clinical, therapeutic, and catheter data were collected from electronic medical records (Medview) and spreadsheets provided by the IVT team. Cost data were obtained from the departments of nursing management, materials assistance, and human resources (HR).

In the final (third) step of measuring the ADC, the direct costs were estimated using criterion values that could be identified, quantified, and measured.^(12)^This cost included the DLC of the professionals directly involved and the inputs used directly in PICC placement.

It is worth noting that there was variation in the object under analysis because each placement presented its particularities with different times (duration) and expenses.

The average wages of these categories related to the study period were used to calculate the DLC of the nurses who placed the catheters and their technical assistants. To obtain the DLC of these professionals, the average time (duration) of the procedure was collected, considering the preparation of materials at the time of placement until completion. The mean time for PICC insertion was 105 minutes (SD=15), ranging from 60 to 120 minutes.

The average unit costs of materials, drugs, and solutions were calculated based on the value of the last acquisition in the year under analysis. To count the materials used, the average unit cost of the input was considered; for drugs and solutions, the average unit cost of an ampule and/or vial was counted. These inputs were then multiplied by the average amount used for each placement. The estimation of input costs was necessary due to the variation in the object of analysis.

The costs of the tests for the years 2019, 2020, and 2021 were used to calculate the costs of imaging tests performed to confirm the PICC tip. The cost of an X-ray examination for each PICC placement eligible for the imaging test was considered, that is, the cost of the X-ray for all insertions using the blind technique and the US-guided technique with X-ray tip confirmation was added to the ADC of the PICC placement. For insertions performed with the real-time tip confirmation technique via intracavitary electrocardiography, the X-ray cost was not added, as it was not used.

The estimated cost was used to measure the ADC. The estimated cost was determined based on the costs of previous periods, without the need for observation during and/or after the production of the service.^(17)^ The variables involved in the ADC of PICC placement as well as the relationship between these variables were defined, with the total ADC being the sum of the average costs.^(12)^

To measure the DLC, the minute value for each professional category (permanent nurse, resident nurse, and technical assistant) and the average unit cost of the DLC were first defined.

The average cost of each professional category was obtained based on an estimate of the average time spent by professionals placing the PICC according to the average unit cost of the DLC. The ADC of the DLC was then obtained as the sum of the average costs of the professionals and the average time dedicated.

To measure the ADC, the variables involved in the direct cost of PICC placement and the relationship between these variables were defined. The total ADC was obtained by summing the average costs, as follows:^(12)^


(1) $$\bar{CdTOTAL}=\bar{\sum_{i=1}^{t}CPt.\;}$$

Since the procedures required different quantities of inputs, we estimated the total ADC for each placement as being composed of four parts: the average cost of materials, medicines/solutions, DLC, and imaging test (X-ray), as follows:


(2) $$\bar{C\left(P_{t}\right)}=\bar{C{\left(P_{t}\right)}_{\text{mat }}}+\bar{C{\left(P_{t}\right)}_{\text{medsol }}}+\bar{+C{\left(Pt\right)}_{\text{dlc}}}\bar{+C{\left(Pt\right)}_{\text{xray }}}.$$

The ADC of the materials was obtained by adding the average costs of each of the materials used, as follows:


(3) $$\bar{C{\left(P_{t}\right)}_{\text{mat}}}=\bar{\sum_{k=1}^{n}C{\text{m}}_{\text{k}}}.$$

The average cost of each material was obtained by multiplying the average quantity of this material by its average unit price, as follows:


(4) $$\bar{\text{C}m_{\text{k}}}=\bar{\text{q}m_{\text{k}}}\cdot \bar{\text{P}mu_{\text{k}}}.$$

By replacing Equation 3 with Equation 4, the following more detailed equation was obtained for the ADC of materials:


(5) $$C{\left(Pt\right)}_{\text{mat }}=\sum_{k=1}^{n}\left(\bar{q}m_{\text{k}}\cdot \bar{\text{P}m_{\text{u}}}\right).$$

The ADC of the medicines/solutions was obtained by adding the average costs of the medicines/solutions used, as follows:


(6) $$\bar{C{\left(Pt\right)}_{medsol\;}}=\sum_{k=1}^{n}\bar{C_{medsol\;}k}.$$

The average cost of each medicine/solution was obtained by multiplying the average quantity of this medicine/solution by its average unit price, as follows:


(7) $$\bar{C_{\text{medsol k}}=}\bar{q_{\text{medsol }k}.}\bar{P_{{\text{medsol u}}_{\text{k}}}.}$$

By replacing Equation 6 with Equation 7, the following more detailed equation was obtained for the ADC of medicines/solutions:


(8) $$\bar{C{\left(P_{t}\right)}_{medsol\;}}=\sum_{k=1}^{n}\bar{(q_{medsol\;}k.}\bar{P_{medsoluk\;})}.$$

The ADC of the DLC was obtained by adding the average costs of each professional category involved in placing the PICC, as follows:


(9) $$\bar{C{\left(P_{t}\right)}_{dlc}}=\sum_{c=1}^{n}\bar{Ch_{c}}.$$

The average cost of each professional category was obtained by multiplying the average time dedicated by professionals to placing the PICC by the average unit cost of labor, as follows:


(10) $$\bar{Ch_{c}}=\bar{t_{c}.Su_{c}.}$$

The ADC of the DLC was calculated by substituting the average values obtained by replacing Equation 9 with Equation 10, as follows:


(11) $$\bar{C{\left(P_{t}\right)}_{dlc}}=\sum_{c=l}^{n}\bar{\left(t_{c}\cdot S_{uc}\right)}\cdot$$

The ADC of the imaging test (X-ray) was obtained by adding the average costs of the imaging tests performed, as follows:


(12) $$\bar{C{\left(P_{t}\right)}_{xray\;}}=\sum_{k=1}^{n}\bar{C_{medsol\;}k}.$$

The average cost of each imaging test was obtained from the product of the average quantity of the imaging test and its average unit price, as follows:


(13) $$\bar{C_{xray\;k}}=\bar{q_{xray\;k}.P}\bar{x_{xry}u_{k}.}$$

By replacing Equation 12 with Equation 13, the following more detailed equation was obtained for the ADC of the imaging test:


(14) $$\bar{C{\left(P_{t}\right)}_{xay}}=\sum_{k=1}^{n}\bar{(q_{xray\;k}P_{xary}}\bar{u_{k})}.$$

By replacing Equation 2 with Equations 5, 8, 11, and 14, the following equation was obtained to determine the ADC of each placement:


(15) $$\begin{array}{c} \bar{C(Pt)}\sum_{k=1}^{n}\bar{(qmk}.\bar{P_{muk})}\\ +\sum_{k=1}^{n}\left(\bar{q_{\text{medsol }}}k\cdot P_{\text{medsol }}u_{k}\right)+\sum_{c=1}^{n}\bar{(t_{c}}\bar{S_{u}})+\\ \sum_{k=1}^{n}\left(\bar{q_{\text{sary }}k.}\bar{P_{xary}uk}\right)\cdot (15) \end{array}$$

Thus, the estimate of the total ADC of PICC placement was obtained by substituting the average values obtained by Equation 15 into Equation 1.^(12)^

The currency used was The Brazilian Real (R$) converted into US dollars (US$) at a rate of US$ 0.19/R$ based on the exchange rate on April 9, 2022, using the online currency converter of the Central Bank of Brazil.

After the data were tabulated, the variables "costs" were presented by observing the minimum and maximum values and calculating the mean, standard deviation (SD), median, and mode.

Statistical, descriptive, and inferential analyses were performed to analyze the results. In addition, the following statistical significance tests (p-value) were employed: the Kolmogorov-Smirnov test, Mann-Whitney U test, and Kruskal-Wallis test.

The profile of the study sample was described, including the variables analyzed and their unfolding. Absolute and relative replications were used. In terms of inferential analysis, the statistical objective was to analyze the independence and prediction among the variables proposed in the scope of the study.

It is worth mentioning that the results of independence between the proposed variables were given through analysis of p-values, where p<0.05 indicated statistical significance. All tests had an alpha error of 5% and a reliability of 95%. Finally, all analyses were obtained using the IBM program Software Statistical Package for the SPSS (version 23) as per the features of Excel^®^ (version 2.016).^(18)^

## RESULTS

During the period analyzed (January 2019 to December 2021), 664 PICC placements were performed in adolescents and adults aged between 15 and 99 years at the institution. A total of 33 placements were considered losses due to incomplete data in the medical records, making it impossible to analyze them; therefore, 631 placements were included in the analysis.

All 631 placements were performed by nurses trained and qualified to place a PICC, in accordance with Resolution 258/2001.^(9)^

Regarding the demographic profile, 207 (32.8%) patients were female while 424 (67.2%) were male, with a mean age of 48.0 years.

The catheter materials were polyethylene, polyurethane, and silicone in 2 (0.3%), 129 (20.4%), and 500 cases (79.2%), respectively, of which 500 (79.2%) were single-lumen and 131 (20.8%) were double-lumen. Of these, 131 were catheter-powered (20.8%), and the remaining 500 (79.2%) were conventional. Sixteen (2.5%) placements were identified using tip confirmation technology with intracavitary electrocardiograms, while 615 (97.5%) used X-rays for tip confirmation.

The main indications for PICC were as follows: 562 (89.1%) for the administration of antimicrobials, 36 (5.7%) for the administration of medications, regarded as vasoactive drugs and sedation, followed by 33 (5.2%) for the infusion of parenteral nutrition.

Regarding the insertion technique, 16 (2.5%) catheters were placed using the US-guided technique with real-time tip confirmation using intracavitary electrocardiography. Additionally, 115 (18.2%) catheters were placed using the US-guided technique, that is, echoguided with tip location via radiography, and 500 (79.2%) catheters were placed by direct puncture (*i.e*., the blind placement technique).

Table 2 illustrates blind, direct puncture placements, which is related to a higher incidence rate of unsuccessful placements, that is, non-progression of the catheter, when compared with US-guided placements.

**Table 2:** Association between the progression or non-progression of catheters and insertion technique

Variable	Catheter progression Yes n (%)	Catheter progression No n (%)	p value
Placement technique	518	113	0.000
Direct puncture	394 (62.4)	106 (16.8)	
US-guided loc tip by X-ray	108 (17.1)	7 (1.1)	
US-guided loc tip by ECG	16 (2.5)	0 (0)	

The catheters were inserted by qualified nurses who belonged to the following categories: nurses, resident nurses, and technical assistants. To calculate the DLC, the average time of PICC placement was set at 105 minutes. In terms of average cost per hour and minute for the professionals involved in PICC placement, the highest cost was observed for the category "nurse," as described in table 3.

**Table 3:** Average cost per hour and minute for nursing professionals involved in peripherally inserted central catheter placement

Professional category	Average cost/hour	Average cost/minute (R$)
Nurse (R$)	32.65	0.54
Resident Nurse (R$)	13.80	0.23
Technical Assistant (R$)	27.51	0.46
Nurse US$*	6.20	0.10
Resident Nurse US$*	2.62	0.04
Technical Assistant US$*	5.22	0.08

Presentations in Brazilian currency: Reais (R$) and American currency: Dollar (US$).

* Exchange rate: US$ 0.19/R$, based on the rate on April 9, 2022, provided by the Central Bank of Brazil.

The catheter kits (epicutaneous catheter + introducer + angulators) presented the highest unit cost and had the greatest impact on cost composition. In particular, the cost of the double lumen, 5 French, polyethylene material catheter kit was substantially high (average unit cost, US$ 220.93), whereas the cost of the epicutaneous, single Lumen, 3 *Fr*, silicone catheter material was considerably low (average unit cost, US$ 54.10) (Table 4).

**Table 4:** Distribution of the types of catheters used, average unit cost, and average total cost

Catheter used in the procedure	Observations n (%)	Average unit cost US$*	Average total cost US$*
Single Lumen 3 French (Fr) silicone	1 (0.15)	54.10	54.10
Single Lumen 3 French (Fr) polyethylene+Kit**	1 (0.15)	220.93	220.93
Single Lumen 5 French (Fr) silicone	498 (78.9)	66.42	33,077.16
Double lumen 4 French (Fr) silicone	1 (0.15)	72.96	72.96
Double lumen 5 French (Fr) polyethylene+ Kit**	1 (0.15)	220.93	220.93
Double lumen 5 French (Fr) polyethylene+ Kit**	129 (20.5)	218.56	28,194.24
Total	631 (100)		61,840.32

* Exchange rate: US$ 0.19/R$, based on the rate on April 9, 2022, provided by the Central Bank of Brazil; **Kit: introducer+US angulators.

Table 5 shows the association between the choice of placement technique/material and the cost of the catheter. Silicone catheters placed by direct puncture had a lower cost than the polyethylene and polyurethane catheters used for US-guided placement, both by X-ray tip location and real-time location with intracavitary ECG.

**Table 5:** Association between the placement technique and material and the catheter cost

Variable	Catheter cost US$ 92.93 to US$185.87	Catheter Cost >$185.87	p value
Placement technique and material	500	131	0.000
Direct Puncture/Silicone, n (%)	500 (79.2)	0 (0)	
US-guided* Loc tip ** by X-ray/ Polyethylene and Polyurethane, n (%)	0 (0)	115 (18.2)	
US-guided* Loc tip** by ECG***/Polyurethane, n (%)	0 (0)	16 (2.5)	

* Ultrasound

** Tip location

*** Electrocardiogram.

Table 6 shows that the cost of PICC placement was US$ 217.14 (SD=75.21), with materials showing the highest cost (US$ 195.39, SD=74.15), justified by the high cost of catheters, followed by the cost with the team's DLC (US$ 20.00, SD=2.22). The total cost related to the 631 insertions, considering the successful and unsuccessful insertions was US$ 137,019.88.

**Table 6:** Distribution of variables involved in peripherally inserted central catheter placement by cost of direct labor and cost of catheters/materials/drugs/solutions

Variables	N	Total US$*		Mean US$*	Median US$*	Mode US$*	SD US$*	Minimum and maximum value US$*
Cost of nursing DLC**	631	6,850.52	10.85		11.30	11.30	0.88	4.58-11.30
DLC** of the technical assistant	631	5,772.70	9.14		7.57	7.54	1.88	7.54-11.37
Total cost of DLC** (nurse and assistant)	631	12,624.41	20.00		18.84	18.84	2.22	12.12-22.68
Total cost of materials/drugs/ solutions and catheter	631	123,297.27	195.39		256.11	256.11	74.15	110.15-306.27
Cost of imaging test	631	1,098.19	1.74		1.78	1.78	0.28	1.74-1.78
Total	631	137,019.88	217.14		280.59	280.59	75.21	124.08-325.76

* Exchange rate: US$ 0.19/R$, based on the rate on April 9, 2022, provided by the Central Bank of Brazil; **Direct labor cost.

## DISCUSSION

The objective of this study was to estimate the direct costs of PICC placement by nurses for hospitalized patients. The results of the descriptive analyses of this study showed that PICC placement occurred predominantly in males (424, 67.2%) and individuals aged 61 years or older, with a lower frequency than in those aged 15 to 29 years. The mean age was 48.0 years, similar to that in other studies on the use of PICC in hospitalized patients.^(19,20)^

The catheters were inserted by permanent and resident nurses with the help of a technical assistant. It is worth mentioning that the residents followed the placement flow recommended by the institution, in which they were only considered qualified after prior training and five placements under the supervision of an experienced professional.

No impact on the time (duration) of the procedure was observed in the placements performed by the residents due to the low number of devices placed by the professionals.

The most commonly used materials were silicone and polyurethane. Additionally, the most commonly used method for catheter tip insertion and confirmation, respectively, were US-guided insertion and confirmation of the tip by radiography, which is in line with the findings of other national studies.^(3,11)^

Among the main reasons for PICC placements was the use of antimicrobials, justified by the vesicant and irritant potential of many drugs, as well as the long treatment period.^(5,21)^

The institution used the ultrasound-guided insertion technique less often, and during the study period, the conventional technique of direct puncture, also known as the blind puncture technique, prevailed. Evidence suggests that echo-guided insertion yields better results than blind insertion. Among the advantages are greater assertiveness due to visualization of vein depth, identification of adjacent vessels and structures, and reduction in the incidence of phlebitis and venous thrombosis, making insertion safer and more effective and reducing procedure time.^(3,22-25)^

Another advanced method is PICC placement with real-time tip location using intracavitary ECG. Studies conducted in China found this technology to be a safe and viable option to improve the success rate of PICC placement in addition to reducing the time of the procedure.^(25-27)^

In terms of the direct costs of PICC, materials were found to be the largest contributor, followed by the DLC of professionals, corroborating the results of other studies on direct costs conducted by nurses in Brazil.^(3,5)^

The mean duration of the procedure was 105 minutes (SD=15), ranging from 60 to 120 minutes. Two single-case studies revealed an average time (duration) of 50 and 46 minutes.^(3,5)^

Studies have shown that an exclusive and qualified team for PICC placement and management reduces procedure time, increases assertiveness, optimizes available resources, thus reducing direct costs as well as providing quality care, patient safety, and comfort.^(28,29)^

Exclusive IVT teams are effective in institutions because they work well and efficiently in the continuing education of professionals and the formulation of institutional protocols based on the best clinical evidence, combined with lower costs.^(5,28,29)^

Cost management is a challenging job for health managers because cost reduction is not only related to resource optimization but is also directly associated with the best care practices developed through training. Studies conducted in Brazil have shown that quality nursing care can reduce the risk of complications inherent to PICC, contribute to the adequate treatment of complications, and reduce costs.^(30,31)^

Although the financial management of a hospital is a very complex task, which includes the acquisition of PICC, it is worth mentioning that the device offers numerous advantages when compared with others, such as the reduction in the number of venous punctures; therefore, saving on materials; reducing patient pain, discomfort, and stress; reducing infiltrations and extravasations and, consequently, reducing expenses associated with the treatment of these adverse events and preservation of the venous network. Moreover, bedside-inserted catheters discard the use of operating or exclusive rooms, and the creation of a protocol for early indication of PICC placement decrease the risk of multiple punctures and increase the possibility of success in the first insertion. Nevertheless, PICC placements may increase the consumption of inputs and generate intangible and tangible costs for both patients and institutions.^(3,19,32)^

This study has some limitations. First, although the study was conducted in 2022 using data from 2019 to 2021, the annual accumulated inflation index of Brazil and the National Broad Consumer Price Index (IPCA - *Índice Nacional de Preços ao Consumidor Amplo*)may have interfered with the average total direct cost presented.

Second, the payment reference for the X-ray test used by the institution, which is financed by the SUS, using the SIGTAP table, has obsolete costs. Hence, there is a potential risk of cost underestimation. Finally, data collection through electronic medical records might have suffered from memory bias shown by the fragility in the recording of some information, such as the exact time of the procedure and number of imaging tests performed by insertion to confirm the catheter tip. In the future researchers should measure the ADC of PICC placement through direct observation of the procedure and conduct economic evaluation studies that can analyze not only the estimated ADC of placement but also the cost-effectiveness of PICC when compared with other devices.

## CONCLUSION

This study estimated the average direct cost of peripherally inserted central catheter placement in Brazil. The findings showed that materials contributed the most to the average direct cost, which was justified by the high unit cost of the catheters and US kits used for echo-guided insertion, followed by the professionals’ direct labor costs.

In particular, the cost of the epicutaneous catheter + introducer, double lumen, 5 Fr, polyethylene was substantially high (average unit cost, US$ 220.93), whereas the cost of the epicutaneous catheter, single lumen, 3 Fr, silicone was considerably low (average unit cost, US$ 54.10).

Measuring the average direct cost of peripherally inserted central catheter placement provides financial visibility for the inputs used. However, the key challenge is promoting lasting changes in the behavior of managers who perform administrative functions in healthcare institutions so that adequate budget management can enhance the efficiency of resource allocation.

Management strategies can be used to guide and sustain the clinical practice of placing and managing peripherally inserted central catheters, such as knowing the flow of the device, time spent by the nurses, the amount of material and human resources involved, and formulating institutional protocols and checklists as well as care and management indicators.

Another measure is to invest in continuing education for the nursing team, who will be directly involved in catheter management, with emphasis on reducing complications related to the inappropriate use of the device and, consequently, minimizing costs.

The results of the current study can be used as tools to support continuing educational actions, strengthen institutional protocols, and prepare professionals and managers to provide safe, harm-free, and quality care as well as to optimize resource allocation and reduce costs for healthcare institutions.
